# Pelviureteric junction obstruction of the ipsilateral kidney caused by hydronephrosis secondary to crossed fused renal ectopia

**DOI:** 10.1002/iju5.12487

**Published:** 2022-06-01

**Authors:** Taiki Kato, Maria Aoki, Koei Torii, Takashi Hamakawa, Hidenori Nishio, Kentaro Mizuno, Yosuke Ikegami, Tetsuji Maruyama, Yutaro Hayashi, Takahiro Yasui

**Affiliations:** ^1^ Department of Urology Nagoya City University East Medical Center Nagoya Japan; ^2^ Department of Urology Nagoya City University West Medical Center Nagoya Japan; ^3^ Department of Pediatric Urology Nagoya City University Graduate School of Medical Sciences Nagoya Japan; ^4^ Department of Nephro‐Urology Nagoya City University Graduate School of Medical Sciences Nagoya Japan

**Keywords:** congenital abnormalities, fused kidney, hydronephrosis, kidney, ureteral obstruction

## Abstract

**Introduction:**

Crossed fused renal ectopia is rare and usually asymptomatic. However, it is associated with urological anomalies.

**Case presentation:**

A 15‐year‐old Japanese boy was transported to our hospital with right abdominal pain and hematuria after a soccer ball hit his right abdomen. Computed tomography revealed right hydronephrosis beyond the center of the body and no left kidney. Percutaneous nephrostomy was performed immediately, and a pyeloplasty was scheduled for 5 months later. Right hydronephrosis was noted to have been caused by left pelvic expansion due to a crossed fused ectopic kidney (secondary to a left pelviureteric junction obstruction). Subsequently, a left dismembered pyeloplasty was performed. Twenty‐four months later, pain and hematuria were absent, and the creatinine level was 1.1 mg/dL. Ultrasonography revealed a shrunken right kidney.

**Conclusion:**

We encountered a unique urological anomaly with crossed fused renal ectopia. Comprehensive anatomical evaluation before surgery is important for maintaining long‐term renal function.

**Abbreviations & Acronyms**
CT: computed tomography
PUJO: pelviureteric junction obstruction
US: ultrasonography
VUR: vesicoureteral reflux

**Keynote message**

Crossed fused renal ectopia is a rare congenital condition that is often associated with various urological abnormalities. A comprehensive anatomical evaluation before surgical intervention is necessary for improved outcomes.

## Introduction

Crossed fused renal ectopia is a rare congenital anomaly. In this, one kidney is present on the side opposite to where the ureter opens into the bladder and is fused below the orthotopic kidney. Most cases of crossed fused renal ectopia are usually asymptomatic; however, they could have multiple associated symptomatic urological anomalies.^1^ Herein, we present a case of a unique urological anomaly associated with crossed fused renal ectopia.

## Case presentation

**Fig. 1:**
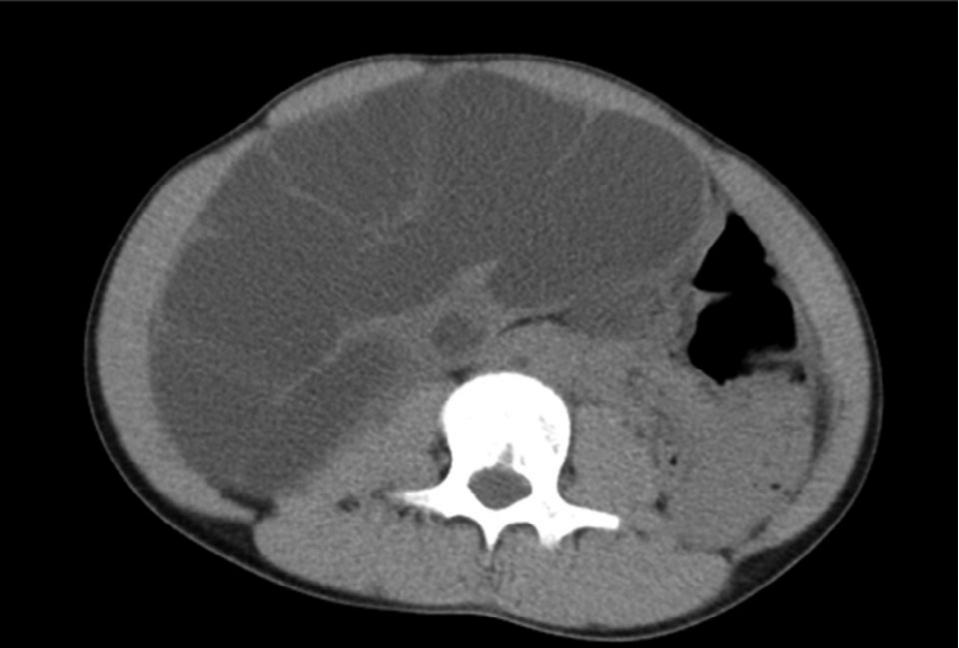
Abdominal CT performed during the first visit. The axial view reveals right hydronephrosis at the center of the body.

**Fig. 2:**
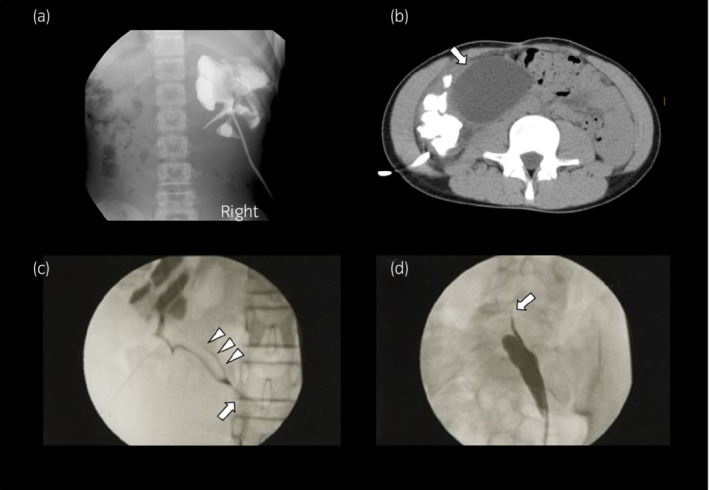
(a) Right antegrade pyelography after nephrostomy reveals shrinking of the renal pelvis and calyx; a right PUJO is suspected. (b) Plain CT performed immediately after right antegrade pyelography reveals a cystic lesion separating from the right pelvis (arrow). (c) Right retrograde pyelography reveals a pelvic filling defect due to a clot (arrowheads) and a PUJO (arrow). (d) Left retrograde pyelography reveals the blind end (arrow) of the ureter in the pelvis.

A 15‐year‐old Japanese boy was transported to our hospital because of right abdominal pain and gross hematuria that occurred after a ball hit his right abdomen during a soccer game. Abdominal US revealed large right hydronephrosis. Laboratory data revealed serum creatinine and hemoglobin levels of 1.48 mg/dL and 12.8 g/dL, respectively. Urinalysis revealed numerous red blood cells. CT revealed right hydronephrosis beyond the center of the spine and did not detect the left kidney (Fig. 1). He underwent an immediate percutaneous nephrostomy with a 12‐Fr catheter and was relieved of the abdominal pain. However, 6 h later, the catheter was obstructed by a blood clot. Therefore, 1 day later, the nephrostomy was dilated by a 16‐Fr catheter. Twelve days later, antegrade pyelography was performed to identify the position and length of the obstruction in the right ureter; the pyelogram obtained showed relieved expansion of the right renal pelvis and calyx and a PUJO (Fig. 2a). Thereafter, CT was performed, which revealed a cyst that was separated from the right renal pelvis adjacent to the right kidney (Fig. 2b). This cyst was speculated to be the cause of the right ureteral obstruction; however, we could not confirm this. Renal dynamic scintigraphy with Tc‐99m diethylenetriaminepentaacetic acid revealed a right renal obstructive pattern and a left non‐functioning kidney (Fig. S1). One month after the nephrostomy, he underwent cystoscopy and bilateral retrograde pyelography. Cystoscopy revealed normal bilateral ureteral orifices, while bilateral retrograde pyelography revealed a right PUJO with a clot‐associated pelvic filling defect (Fig. 2c) and the blind end of the left ureter in the pelvis (Fig. 2d). Accordingly, he was diagnosed with a right PUJO and a left hypoplastic kidney. He was scheduled for a right pyeloplasty 5 months after his first visit.

**Fig. 3:**
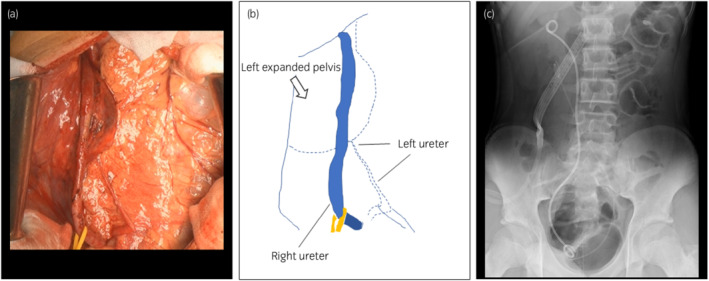
(a, b) Images illustrating the operation. The right ureter had no stenosis and was compressed by the expanded left renal pelvis. The left ureter was thread‐like, and the patient was diagnosed with a left PUJO. (c) A kidney–ureter–bladder examination performed after the operation. The patient was diagnosed with a left crossed and fused ectopic kidney.

**Fig. 4:**
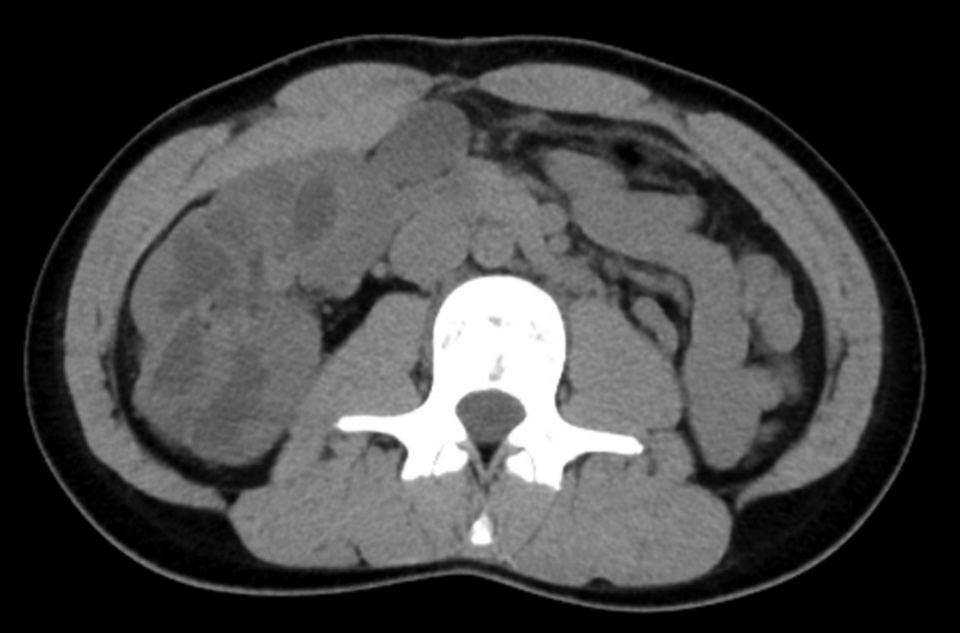
Abdominal CT performed 12 months after the left pyeloplasty. The axial view reveals shrinking of the right renal pelvis and calyx.

Under general anesthesia, a right ureteral stent was placed prior to the pyeloplasty procedure. The patient was placed in a supine position, and a ventral incision was made. The ascending colon was mobilized by incising the lateral peritoneum, and the right ureter was identified. The right ureter at the ureteropelvic junction was peeled from the surrounding tissues, and no stenosis was observed in it. The left ureter was thread‐like over a length of 4 cm, and the left renal pelvis was cystically expanded (Fig. 3a,b). A left dismembered pyeloplasty was then performed, and a ureteral stent was placed in the left ureter (Fig. 3c). The operation revealed a left crossed and fused ectopic kidney; left pelvic expansion secondary to right hydronephrosis‐associated left PUJO was also observed. Twelve months after the operation, CT revealed shrinkage of the right renal pelvis and calyx (Fig. 4). Twenty‐four months later, there was no recurrence of abdominal pain and gross hematuria, and the serum creatinine level was maintained at 1.1 mg/dL.

## Discussion

Crossed renal ectopia is a rare congenital malformation, with an incidence of 1 in 7500 autopsies and 1 in 14 000 in pediatric admissions.^1^ By definition, both kidneys are present unilaterally when the ureter of the crossed kidney opens at where the ureter enters the bladder on the contralateral side. Left‐to‐right ectopia is more common.^1^ More than 90% of the crossed kidney fuses with the orthotopic kidney.^2^ Patients with crossed fused renal ectopia are usually asymptomatic; however, urinary tract infections in infants or abdominal pain, hematuria, hypertension, and renal failure in school‐aged children and adolescents have been observed.^3^ Patients with crossed renal ectopia have highly associated genital and urological anomalies,^4^ such as the VUR, hydronephrosis, cryptorchidism, and hypospadias.^1^, ^2^ VUR was reportedly the most frequently associated urological anomality (37.5%), followed by PUJO.^2^ Surgery and management of patients with crossed fused renal ectopia are guided by the urological abnormalities, residual renal functions, and symptoms; the patients should be treated on a case‐to‐case basis.

This report presents the case of a unique urological anomaly involving orthotopic hydronephrosis caused by an expanded pelvis, which in turn was caused by a crossed fused ectopic kidney secondary to PUJO. Reportedly, 50% of the kidneys with PUJO were non‐functioning^2^ in patients with crossed fused renal ectopia. Furthermore, 37% of the patients of renal ectopia reportedly developed hydronephrosis due to PUJO.^5^ In our case, PUJO may have led to the expansion of the left pelvis, which compressed the right ureter. Crossed fused renal ectopia is associated with a high incidence of many anomalies of both the crossed and orthotopic kidneys; hence, it is important to reveal the accurate anatomical delineation on an individual basis. US is an initial imaging modality that can reveal the absence of a kidney from its normal position and hydronephrosis. However, it is sometimes difficult to detect crossed ectopic kidneys by US.^3^ Radionuclide scanning is preferred as the subsequent procedure, because it can provide information on the renal function and drainage patterns with less radiation exposure.^2^ Contrast‐enhanced CT provides accurate information on the anatomical structure and vascular supply; this helps in the surgical planning and in the screening for urinary stones, tumors, and other congenital anorectal or vertebral anomalies.^6^ In this case, CT after antegrade pyelography was helpful for detecting the cyst that had separated from the right renal pelvis; this cyst was intraoperatively revealed to be a left pelvic expansion. Comprehensive anatomical and renal functional evaluations of patients with symptomatic crossed fused renal ectopia are necessary for appropriate surgical intervention or management. Long‐term follow‐up is also required to preserve the postoperative renal function.

## Conclusion

We encountered a case of a unique urological anomaly with crossed fused renal ectopia, in which orthotopic hydronephrosis was caused by PUJO of the crossed fused ectopic kidney. A comprehensive preoperative anatomical evaluation is important for maintaining long‐term renal function.

## Author contributions

Taiki Kato: Conceptualization; data curation; formal analysis; investigation; methodology; writing – original draft. Maria Aoki: Writing – review and editing. Koei Torii: Writing – review and editing. Takashi Hamakawa: Writing – review and editing. Hidenori Nishio: Writing – review and editing. Kentaro Mizuno: Data curation; supervision; writing – review and editing. Yosuke Ikegami: Writing – review and editing. Tetsuji Maruyama: Supervision. Yutaro Hayashi: Supervision. Takahiro Yasui: Supervision.

## Conflict of interest

The authors declare no conflict of interest.

## Approval of the research protocol by an Institutional Reviewer Board

This study was approved by the Nagoya City University East Medical Center Institutional Review Board (approval no.: 21‐04‐340).

## Informed consent

Written informed consent was obtained from the patient and their guardian for the publication of this case report and accompanying images.

## Registry and the Registration No. of the study/trial

Not applicable.

## Supporting information


**Fig. S1.** (a, b) Renal dynamic scintigraphy with Tc‐99m diethylenetriaminepentaacetic acid with Lasix showed 99.8% right renal split function (green line) with cumulative curve of the right kidney and non‐functional left kidney (white line).
