# Supplementary figures and images for: Pelviureteric junction obstruction of the ipsilateral kidney caused by hydronephrosis secondary to crossed fused renal ectopia
Source: IJU Case Rep. 2022 Jun 1;5(5):354–7. doi: 10.1002/iju5.12487 (PMC9436682; doi:10.1002/iju5.12487)

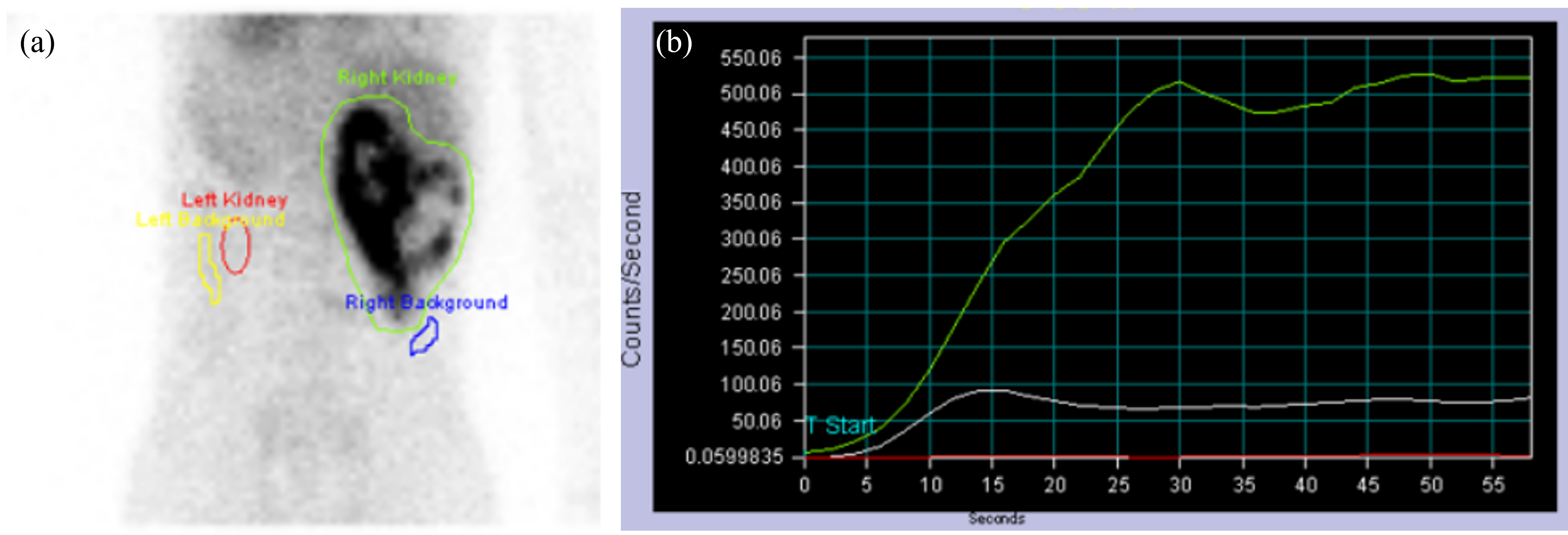

Supplement: Supplementary file 1 — Fig. S1. (a, b) Renal dynamic scintigraphy with Tc‐99m diethylenetriaminepentaacetic acid with Lasix showed 99.8% right renal split function (green line) with cumulative curve of the right kidney and non‐functional left kidney (white line). [file IJU5-5-354-s001.tif]
